# Orthopedic surgery assisted by mixed reality technology: systematic review

**DOI:** 10.3389/fsurg.2026.1770605

**Published:** 2026-03-25

**Authors:** Lucia Salazar Carrasco, Juan A. Sánchez-Margallo, Francisco M. Sánchez-Margallo

**Affiliations:** 1 Bioengineering and Health Technologies Unit, Jesús Usón Minimally Invasive Surgery Centre, Cáceres, Spain; 2 Scientific Direction, Jesús Usón Minimally Invasive Surgery Centre, Cáceres, Spain

**Keywords:** extended reality, mixed reality, MR-assisted surgery, orthopedic surgery, surgical planner

## Abstract

Mixed Reality (MR) in the healthcare field provides innovative tools for training, diagnosis, and treatment. It allows professionals to practice surgical procedures in a controlled, risk-free environment, improving skill and reducing error margins, thus offering a more interactive and personalized option for patients. MR aids in the detailed visualization of bone structures and tissues, enabling more precise planning. To conduct this systematic review, a rigorous and structured approach was followed to identify, evaluate, and synthesize relevant studies on the application of MR in orthopedic surgery. The following keywords and combinations were used: “Extended Reality”, “Mixed Reality”, “Orthopedic Surgery”, and “Surgical Planner” in PubMed and Web of Science over the last 10 years. Studies included in this review met the following exclusion criteria: articles that did not use these technologies, studies on other types of surgery, or were review articles. MR has opened new possibilities in the field of surgery, transforming both the training of medical professionals and the surgical interventions themselves. While there are still challenges in its widespread implementation, the benefits it offers in terms of precision, personalization, and efficiency are undeniable. As this technology continues to evolve, it is expected to have an even greater impact on the medicine of the future, making surgeries safer and improving patient outcomes.

## Introduction

Orthopedic surgery encompasses the diagnosis and treatment of musculoskeletal injuries and disorders through surgical interventions aimed at restoring function and alleviating pain. However, it encounters several significant limitations, including the intricate visualization of complex internal structures in real time, the precision required for implant or prosthesis placement, and the imperative to minimize surgical errors (1). Surgical assistance can effectively address these challenges by overlaying 3D patient images onto their bodies during surgery (2). This technique facilitates precise guidance, optimizes surgical planning, reduces operating times, and diminishes the incidence of complications.

Over the past decade, Extended Reality (XR), including virtual reality (VR), augmented reality (AR), and mixed reality (MR), has revolutionized various sectors, including the field of surgery. One of the most promising innovations in the field of surgery has been the incorporation of mixed reality (MR), a technology that merges AR and VR in order to improve both surgical precision and the training of medical professionals (3). MR is becoming a key tool in various processes within the operating room, and its impact is increasingly palpable in medical practice.

MR refers to the combination of elements from the physical world and the virtual world, allowing both to interact in real time. Unlike AR, which superimposes virtual objects onto a real environment, MR not only displays these objects, but also allows them to interact more deeply and realistically with the environment. This approach offers surgeons the ability to superimpose medical images, such as 3D models of organs or anatomical structures, directly onto the patient's body.

Mixed reality offers significant advantages in the surgical field, such as more accurate preoperative planning using interactive 3D models based on the patient's medical images, allowing for the design of personalized strategies and the rehearsal of complex procedures in advance (4). During surgery, it provides real-time assistance by superimposing diagnostic images onto the surgical field, improving accuracy and reducing errors (5). It also revolutionizes medical training by enabling safe practice in realistic virtual environments, and promotes remote collaboration, allowing experts to assist other surgeons remotely through immersive platforms.

In the field of orthopedic surgery, the utilization of MR enables surgeons to comprehensively visualize bone structures and soft tissues in three dimensions. This capability significantly enhances the precision of surgical planning and execution (6). Moreover, MR integrates real-time medical images, thereby facilitating informed decision-making processes. Furthermore, the field of medical training is witnessing an increasing demand for immersive training environments. Mixed reality technology offers such a safe setting for surgeons in training to practice techniques without endangering patients (7).

However, its implementation presents several challenges, such as the high cost of equipment and technology, which may limit its accessibility in some clinical settings. In addition, integrating this technology into the clinical workflow requires specialized training for professionals. Compatibility issues between MR systems and existing medical infrastructures may also arise, potentially resulting in technical and operational challenges.

Consequently, the primary objective of this study is to comprehensively analyze the current state of mixed reality technology application within the domain of orthopedic surgery. This analysis will elucidate the potential benefits and limitations that necessitate attention in the subsequent development of this technology.

## Materials and methods

### Search strategy

A comprehensive literature search was conducted in the PubMed and Web of Science databases to identify relevant studies on the application of mixed reality in orthopedic surgery. This systematic review was designed according to the Preferred Reporting Items for Systematic Reviews and Meta-Analysis (PRISMA) statement for reporting systematic reviews and meta-analyses (8). The following search string was used for the scientific databases consulted:

(“mixed reality”) AND [(orthop*) AND (surg*)]

The strategy was limited to the last ten years, restricted to full-text articles, and conducted in English. Duplicates were removed before the screening and eligibility assessment process.

### Selection criteria

The research question of this systematic review was defined according to the PICOS framework. The population (P) included human patients undergoing orthopedic surgical procedures. The intervention (I) was the intraoperative use of MR technologies during real clinical orthopedic surgeries. Comparator(s) (C), when applicable, included conventional surgical techniques performed without MR or using alternative navigation or visualization technologies. Outcomes (O) of interest comprised any reported clinical outcomes. Eligible study designs (S) included randomized controlled trials, cohort studies, observational studies, and clinical case series or reports involving real surgical interventions.

Taking into account the aforementioned research question, a series of inclusion and exclusion criteria were established to select the studies that best fit the objectives of this review. Inclusion criteria comprised studies involving human orthopedic surgery patients in which MR technologies were implemented during actual surgical procedures performed in real clinical settings, including both intraoperative applications and postoperative follow-up. Comparison studies could include surgeries performed without MR or with other technological tools. Outcomes of interest focused primarily on accuracy, operative time, and clinical outcomes.

Studies that focused exclusively on preoperative planning phases, used animal or cadaveric models, or employed augmented reality or virtual reality instead of mixed reality were excluded. Similarly, studies limited to training, validation of solutions without surgical application, or simulation in phantoms were not considered. In addition, review articles, studies without full-text access, and those related to other surgical specialties were excluded from the analysis.

### Evaluation of methodological quality

To assess the methodological quality of this systematic review, a structured PRISMA checklist based on methodological assessment standards was applied (8). This checklist included items related to study design, internal validity, risk of bias, and clarity in the presentation of results.

To assess the bias of the included studies, we used the RoB2 and ROBINS-I tools. For randomized studies, version 2 of the Cochrane Risk of Bias assessment (RoB 2.0) tool was used (9). This tool assesses six criteria: the randomization process, deviations from planned interventions, outcome measurement, missing outcome data, selection of reported outcomes, and overall bias. Each criterion is classified into one of three categories: high risk, some concern, and low risk. For non-randomized studies, the Risk of Bias in Non-Randomized Intervention Studies (ROBINS-I) assessment tool was used (10). This tool assesses several areas, including pre-intervention, confounding factors, participant selection, classification of interventions, deviations from planned interventions, missing data, and outcome measurement. Similarly, the ROBINS-I assessment was divided between studies involving more than one patient and case reports. In case of discrepancies in the assessment of the criteria, an external expert assessor was consulted.

### Data extraction

The following data were extracted from each study included in this review: the type of orthopedic specialty and surgery performed, the number of participants, the mixed reality (MR) device used, the software applied for medical image segmentation and 3D reconstruction, the MR platform or interface used during surgery, and the main clinical and surgical outcomes reported.

Regarding surgical data, extracted variables included the type of orthopedic procedure (e.g., total hip or knee arthroplasty, spinal fixation, percutaneous kyphoplasty, pedicle screw placement, or limb-preserving surgery), the anatomical site involved, operative time, and intraoperative accuracy when reported.

The software used for medical image processing (e.g., image segmentation and 3D rendering) encompassed tools designed for processing DICOM images and generating 3D models prior to surgery. These tools were used to segment CT or MRI images and export patient-specific 3D anatomical data to the MR platform. The MR platform referred to the operating environment or visualization system that enabled surgeons to interact with holographic or 3D anatomical models during real surgical procedures.

All extracted data were systematically recorded in a data extraction table specifically designed for this review to ensure consistency, transparency, and reproducibility in the analysis process. Numeric data have been presented as percentages.

Due to the marked heterogeneity in study design, surgical indications, MR platforms, and reported outcome measures, it was not considered methodologically appropriate to perform a quantitative meta-analysis. Therefore, the results were synthesized using a structured qualitative approach.

## Results

### Selection of articles

**Figure 1:**
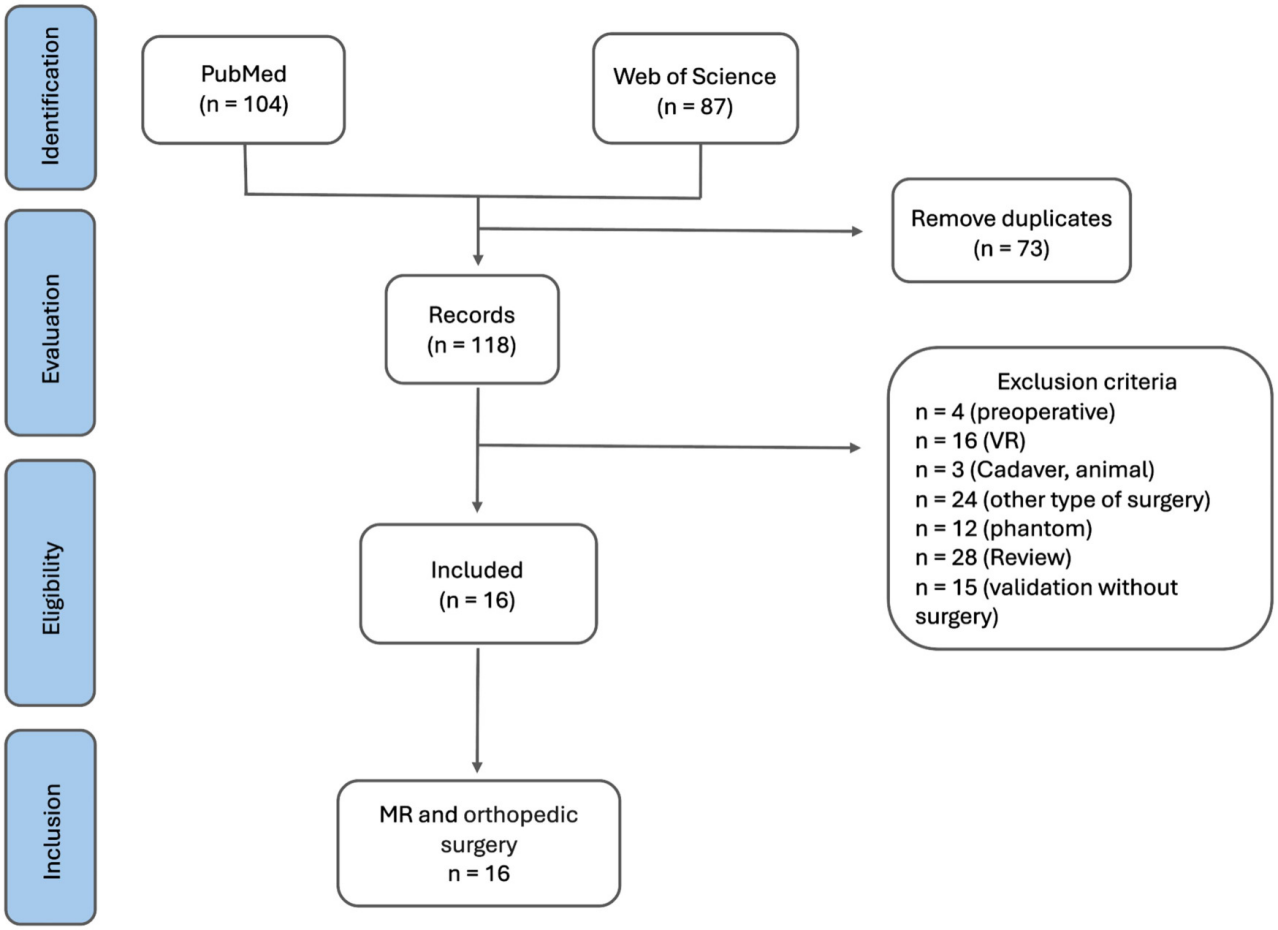
PRISMA flow diagram. Diagram showing the manuscript selection process in accordance with PRISMA guidelines.

A total of 191 were identified, 104 in PubMed and 87 in Web of Science databases. Based on the analysis of the title and abstract of each article, 172 studies were excluded due to duplication (73), wrong subject (20), non-clinical studies (15), studies in a different surgical discipline than the one aimed at this review (24), review studies (28), and studies without clinical application in human patients (15). Only clinical studies involving real surgical interventions in human patients were included. Non-clinical studies (phantom, cadaveric, simulation, engineering, or workflow demonstrations) were excluded because they do not provide patient outcomes or allow risk-of-bias assessment. After comprehensive analysis of the resulting studies, 16 studies were selected (Figure 1).

Results are presented as a systematic qualitative synthesis of clinical evidence, structured by anatomical region, surgical indication, and technological workflow. Table 1 summarizes the findings of the systematic review, organizing the included studies according to the orthopedic region addressed (hip, spine, knee, foot, and shoulder). The extracted data were classified into four primary domains: the mixed reality device used, the preoperative planning/segmentation software, the intraoperative guidance technology, and the patient cohort involved in each study.

**Table 1:** Clinical applications of mixed reality in orthopedic surgery. MR, mixed reality. For each included study, the table reports the orthopedic specialty, surgical procedure, sample size (MR/conventional), MR hardware device (head-mounted display), preoperative image segmentation and planning software, and the intraoperative MR visualization or guidance platform used during surgery. Proprietary and institution-specific software solutions are reported using the terminology provided by the original authors.

Study	Specialty	Procedure	Patients (MR/Conv)	MR device	Preoperative segmentation/planning software	Intraoperative guidance system
Ivanov et al. (11)	Hip	Hip pathology surgery	8	HoloLens™ 2	3D Slicer	Unity
Lu et al. (12)	Spine	Lumbar/pelvis/C1–2/T12	6	HoloLens™ 2	StarCloud workstation	HoloLens™
Zhang et al. (13)	Foot	Total Knee Replacement (TKR Unilateral)	6	HoloLicyo™	Artisight 3D STS	HoloLicyo™
Wang et al. (7)	Foot	Anterior cruciate ligament (ACL single-bundle)	21/23	HoloLens™ 2	Mimics	Msix file
Li et al. (14)	Spine	Lumbar fracture	7	HoloLens™ 2	Mimics v10.1	MITINS
Aoyama et al. (6)	Spine	Spinal cord tumors	5	HoloLens™ 2, Magic Leap™ 1, Oculus Quest™	Ziosation 2 Blender	Holoeyes
Su et al. (15)	Knee	Right total knee	1	HoloLens™ 2	Mimics v19.0	MIDIVI Cloud
Gu et al. (16)	Hip	Pelvic screw placement	25/25	HoloLens™ 2	Midivi 3D	ML System + HoloLens™ 2
Kopriva et al. (17)	Shoulder	Total shoulder (TSA)	97	HoloLens™ 2	Blueprint	–
Legosz et al. (18)	Hip	Complex revision	1	HoloLens™ 2	CT thresholding	DICOM viewer
Wei et al. (19)	Hip	Percutaneous kyphoplasty	20/20	HoloLens™ 2	M3D	Medivi MR
Aoyama et al. (20)	Spine	Cervical/thoracic/lumbar	49	HoloLens™ 2, Magic Leap™ 1,	Ziosation 2 Plus	Holoeyes MD
van der Putten et al. (21)	Knee	Complex total knee replacement (TKA)	1	HoloLens™ 2	–	Dynamics 365 + HoloLens™ 2
Harel et al. (22)	Spine	Lumbosacral pediatric screws	19	HMD glasses	M3D	Xvision-Spine™
Heimann et al. (23)	Hip	Total Hip Replacement (THA)	40	HoloLens™ 2	HipInsight™	HipInsight™
Dilbone et al. (24)	Hip	Acetabular placement	79	HoloLens™ 2	HipInsight™	HipInsight™

Taken together, these studies demonstrate that MR has been preferentially deployed in orthopedic surgeries that require strict spatial orientation, notably hip arthroplasty and pelvic fixation, as well as spinal procedures involving multi-axial screw trajectories or tumor resections (Table 1). In contrast, applications in knee and foot surgery remain exploratory and case dependent. The technological ecosystem is dominated by Microsoft HoloLens™ 2, not due to proven clinical superiority, but because of platform maturity and integration with established segmentation workflows (Mimics, HipInsight, Ziostation). Importantly, the shift from pure visualization to workflow-integrated MR systems in recent high-volume series suggests a gradual maturation of the field. However, overall evidence remains limited by small sample sizes and heterogeneous intraoperative guidance implementations, indicating that clinical adoption is still in an early translational stage rather than widespread routine practice.

Hip procedures represent the largest group (37.5%), followed by spine surgery (31.25%). MR was less frequently applied in knee (12.5%) and foot procedures (12.5%), while shoulder surgery accounted for a single study (6.25%). These proportions highlight that most current initiatives are concentrated in high-complexity anatomical regions where spatial orientation and 3D visualization are clinically relevant. The majority of applications were associated with implant positioning and trajectory guidance. Reports of MR use in ACL reconstruction, complex arthroplasty or other peripheral procedures were limited. In the case of hip procedures, they commonly involved acetabular component positioning, percutaneous screw placement and total hip arthroplasty, while spine applications comprised cervical, thoracic and lumbar interventions, including tumor resection and pedicle screw placement.

In most studies, MR was applied to small or moderate cohorts, typically ranging from single pilot cases to fewer than 30 patients, reflecting its use in early clinical evaluations. Only two studies reported balanced MR/Conventional groups, allowing direct comparison of outcomes. Overall, the number of patients treated under MR remains limited, suggesting that current evidence is still preliminary and concentrated in feasibility or proof-of-concept settings.

With regard the device used, the most frequently reported system was HoloLens™ 2 (Microsoft Corporation, Redmond, WA, USA), accounting for 70% of all reported cases. Magic Leap™ 1 (Magic Leap, Inc., Plantation, FL, USA) appeared in 10% of the studies, while Oculus Quest (Meta Platforms, Inc., Menlo Park, CA, USA), HoloLicyo™ (HoloLicyo Co., Ltd., Beijing, China), and HMD-based systems such as Xvision-Spine™ (Augmedics Ltd., Arlington Heights, IL, USA) each represented 5%. These proportions confirm that the clinical landscape of MR remains largely dominated by the HoloLens™ 2 ecosystem, with other head-mounted displays still at an exploratory or experimental stage of implementation in orthopedic surgery.

The most frequently employed preoperative software was M3D Digital Medical Software (M3D Co., Ltd., Shanghai, China) and the HipInsight™ System (Cleveland Clinic Innovations, Cleveland, OH, USA), each used in 12.5% of the included studies. Other software platforms were reported in single studies (6.25% each), including 3D Slicer 4.13 (25), Mimics Medical (Materialise NV; Leuven, Belgium), StarCloud Workstation (StarCloud Technology Co., Ltd., Shanghai, China), Blueprint (Stryker Corporation, Kalamazoo, MI, USA), ZioStation2 Plus (Ziosoft, Inc., Tokyo, Japan), and Midivi 3D (Midivi Technology Co., Ltd., Beijing, China). Collectively, these programs illustrate a fragmented technological landscape, with most institutions relying on general-purpose segmentation software or manufacturer-specific solutions tailored to their MR workflow.

As intraoperative guidance systems, the analyzed studies included Holoeyes™ (Holoeyes Inc., Tokyo, Japan) and the HipInsight™ System, each reported in 12.5% of the studies. Other systems appeared in isolated reports (6.25% each), including MiDIVI Cloud (Midivi Technology Co., Ltd., Beijing, China), Microsoft Dynamics 365 Remote Assist (Microsoft Corporation, Redmond, WA, USA), and the Xvision-Spine™ System (Augmedics Ltd., Arlington Heights, IL, USA). This distribution highlights that most intraoperative visualization environments are institution-specific or manufacturer-integrated, with the Holoeyes™ and HipInsight™ solutions.

**Figure 2:**
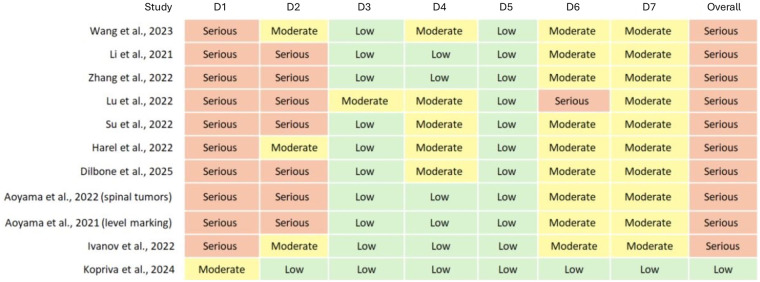
Risk of bias was evaluated using ROBINS-I, with seven domains (D1 -confounding-, D2 -selection of participants-, D3 -classification of interventions-, D4 -deviations from intended interventions-, D5 -missing data-, D6 -measurement of outcomes-, and D7 -selection of reported results-) rated as low, moderate, serious, or critical risk of bias. The following clinical studies were analyzed: Wang et al. (7); Li et al. (14); Zhang et al. (13); Lu et al. (12); Su et al. (15); Harel et al. (22); Dilbone et al. (24); Aoyama et al. (spinal tumors) (6); Aoyama et al. (level marking) (20); Ivanov et al. (11); Kopriva et al. (17).

Concerning the risk of bias analysis using the ROBINS-I tool, most studies exhibited a serious risk of bias due to confounding (D1), largely attributable to the absence of randomization and heterogeneity in surgical procedures, patient populations, and devices used. Similarly, selection of participants (D2) was frequently rated as serious or moderate, reflecting potential inclusion bias and lack of standardized recruitment criteria (Figure 2). In contrast, the domains related to classification of interventions (D3), deviations from intended interventions (D4), and missing outcome data (D5) were generally assessed as low risk, given the clear procedural descriptions and complete follow-up in most reports. Outcome measurement (D6) and selection of reported results (D7) were predominantly rated as moderate, due to variations in outcome assessment methods and reporting practices. Collectively, the evidence indicates a serious overall risk of bias, primarily driven by confounding and participant selection.

**Figure 3:**
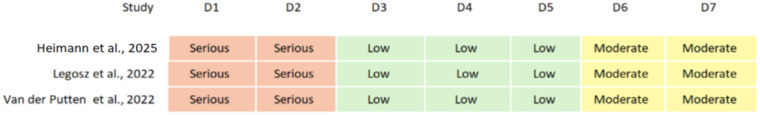
ROBINS-I risk of bias assessment for case reports. Studies included: Heimann et al. (23), Legosz et al. (18), van der Putten et al. (21). Single-patient designs cannot establish causal inference and are therefore classified as Critical overall risk regardless of domain-level performance.

**Figure 4:**
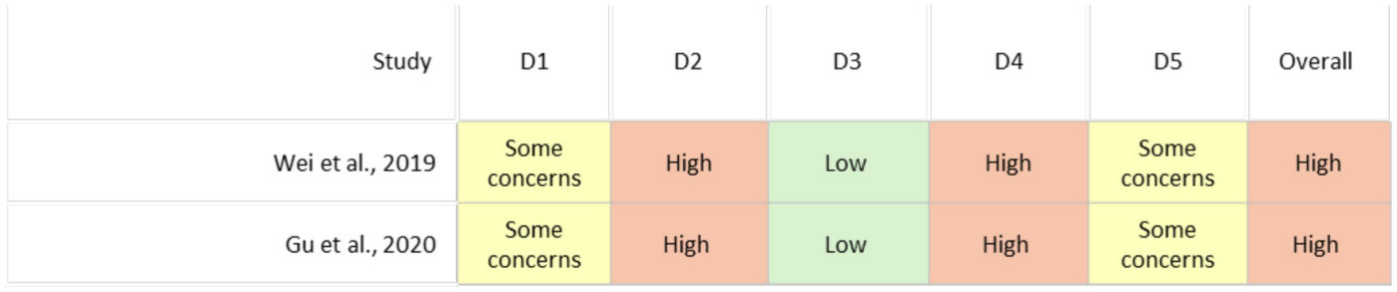
Methodological bias of randomized studies was assessed using the Cochrane RoB 2.0 tool, which evaluates five domains related to internal validity (D1 (randomization process), D2 (deviations from intended interventions), D3 (missing outcome data), D4 (measurement of the outcome), and D5 (selection of the reported result)). Each domain was categorized as Low, Some concerns, or High risk of bias. The assessment was performed for the following studies: Wei et al. (19) and Gu et al. (16).

Observational and cohort studies were evaluated using the ROBINS-I framework (Figure 3). Although most studies demonstrated low risk in the procedural domains (D3–D5), they were generally classified as having a *serious to moderate overall risk of bias*, primarily due to confounding factors and participant selection (D1–D2). Outcome measurement (D6) and selective reporting (D7) were most frequently rated as *moderate*, reflecting heterogeneity in imaging accuracy of metrics and variable reporting standards. Despite these limitations, recent studies showed a trend toward improved methodological rigor, with clearer inclusion criteria, standardized intervention protocols, and more consistent outcome assessment. Overall, the current body of evidence indicates that while mixed reality applications in orthopedic surgery are methodologically feasible, further improvements in study design and bias control are required to strengthen the reliability and generalizability of findings.

The two randomized controlled trials included in this review were assessed using the Cochrane Risk of Bias 2.0 (RoB 2) tool (Figure 4). Both studies demonstrated some concerns regarding the randomization process (D1) and high risk of bias related to deviations from intended interventions (D2) and outcome measurement (D4), primarily due to the lack of blinding among surgeons and evaluators. The missing outcome data (D3) domain was considered low risk, while selection of the reported results (D5) showed some concerns owing to incomplete pre-registration and reporting inconsistencies. Overall, both RCTs were judged to present a high overall risk of bias, highlighting the need for improved randomization transparency, adherence to predefined protocols, and the incorporation of blinded outcome assessment in future clinical trials.

## Discussion

This review analyses the current evidence from clinical studies on the use of mixed reality (MR) in orthopedic surgery, emphasizing its translational maturity, technological barriers, and future clinical potential. The synthesis highlights how MR has transitioned from experimental visualization to an emerging intraoperative tool that enhances surgical accuracy and spatial perception.

MR is increasingly recognized as a surgeon-centered visualization aid rather than a direct replacement for conventional navigation systems. Across the included studies in this review, MR demonstrated consistent clinical value in procedures requiring high spatial accuracy and complex anatomical interpretation. This was particularly evident in hip arthroplasty and acetabular placement, where holographic implant alignment reduced dependence on fluoroscopy and improved the surgeon's understanding of pelvic orientation (12, 22, 23). In spinal surgery, MR supported multilevel planning, screw trajectory definition, and tumor resection, domains where conventional screen-based navigation demands repeated mental mapping and elevates cognitive load (12, 22, 23).

The concentration of MR applications in high-precision procedures reflects a pragmatic pattern of clinical adoption. Surgeons appear to reserve MR for operations where translating preoperative imaging data into intraoperative execution is most demanding. Contrary to the perception of MR as a universal solution, its application in knee, shoulder, and foot surgery remains exploratory (7, 13, 15). These findings suggest that MR's benefits are procedure-specific and particularly pronounced in anatomically complex interventions that demand precise spatial navigation.

Most MR implementations were performed in small or moderate cohorts, consistent with the early stage of clinical integration. Only a few studies provided direct comparisons with conventional navigation, limiting conclusions regarding clinical superiority (17, 19). Among the analyzed literature, hip and spine procedures accounted for nearly 70% of all MR applications (22–24). Hip surgeries primarily involved acetabular alignment, percutaneous screw guidance, and total hip arthroplasty (23, 24), while spinal cases included cervical, thoracic, and lumbar operations, as well as kyphoplasty and tumor resections (6, 22). In contrast, knee, foot, and shoulder applications were infrequent and largely exploratory (7, 13, 15, 17, 21). This uneven distribution underscores that MR adoption is driven primarily by surgical complexity and intraoperative visualization requirements, with substantial potential for future validation in peripheral orthopedic fields.

Within hip surgery, MR was most frequently applied for implant positioning and navigation assistance (23, 24). Acetabular component alignment and total hip arthroplasty (THA) emerged as the most common applications, emphasizing MR's clinical relevance for procedures demanding millimetric precision (23, 24). Additionally, MR-assisted percutaneous pelvic screw placement enabled improved visualization of trajectories and reduced fluoroscopic exposure (12, 23, 24). Complex revision surgeries also benefited from MR-based holographic guidance for preoperative planning and intraoperative orientation.

The Microsoft HoloLens™ 2 was the predominant device, consistently utilized in both preoperative planning and intraoperative guidance. The first-generation HoloLens™ and other head-mounted displays, such as Magic Leap™ or customized smart glasses, were reported only sporadically. This dominance likely reflects the HoloLens™ 2's superior clinical usability, mature software ecosystem, and seamless integration within surgical workflows.

Segmentation and preoperative planning predominantly relied on imaging-based software such as Mimics and 3D Slicer, often combined with custom visualization modules for trajectory guidance and implant alignment. Proprietary or manufacturer-specific systems appeared only in isolated cases (11, 12, 14). This pattern highlights the current need for flexible, general-purpose segmentation workflows that can be easily integrated into MR environments.

Intraoperative guidance was mainly delivered through MR-native holographic overlays, allowing direct visualization of anatomical structures, implant trajectories, or screw paths within the surgical field. Systems such as HipInsight™ or HoloRender™ offered real-time holographic alignment tools, while Unity-based or institution-specific platforms enabled tailored visualization. Conventional navigation systems were used only occasionally and primarily as complementary tools (17, 19). Overall, these trends illustrate an early shift toward immersive, surgeon-centered navigation ecosystems that minimize cognitive fragmentation during surgery.

Despite promising results, the integration of MR into everyday surgical practice remains hindered by several technical limitations. Optical see-through devices, such as head-mounted displays, are constrained by a limited field of view, hologram drift during head movement, and the need for manual calibration. These limitations are manageable in fixed surgical fields but problematic in dynamic environments such as total knee arthroplasty.

Furthermore, current MR workflows depend heavily on advanced segmentation and 3D reconstruction software. Most studies required CT-based modeling with tools like Mimics, 3D Slicer, or proprietary platforms, creating barriers related to imaging access, software training, and engineering support. Finally, intraoperative MR navigation still lacks integration with real-time tracking, haptic feedback, and automated registration, rendering it a passive visualization aid rather than an interactive, closed-loop navigation system (14).

The current evidence base presents several notable methodological limitations. Most studies were conducted in early-adopter, single-center settings with high surgical expertise, introducing adoption and center-related bias and limiting external validity. Sample sizes were generally small, and the predominance of non-randomized designs restricted the available evidence to feasibility and early clinical applicability rather than comparative effectiveness. In addition, substantial heterogeneity was observed across surgical procedures, outcome measures, and MR implementations, further constraining data synthesis and interpretation. MR was predominantly applied in complex or revision cases, which may inherently amplify its perceived benefit compared with standard procedures. Consistently serious or high risk of bias identified across many studies using ROBINS-I and RoB 2.0 underscores that current findings should be interpreted as preliminary and exploratory, and do not yet support conclusions regarding clinical effectiveness, superiority, or long-term patient-centered outcomes.

Outcome reporting also remains heterogeneous. While several studies measured technical, clinical, ergonomic, or radiation-related outcomes, only a minority assessed long-term metrics such as functional recovery, complication rates, or revision outcomes. Consequently, existing data support the technical feasibility of MR but not its clinical superiority. Additionally, the reliance on proprietary devices and non-standardized workflows constrains reproducibility and scalability across institutions.

Future research must evolve from feasibility-level demonstrations toward evidence-based clinical validation. Prospective randomized trials and multicenter comparative studies are essential to determine whether MR can improve surgical accuracy, reduce complications, and enhance implant survival. Development of interoperable and standardized MR ecosystems, integrating preoperative imaging, intraoperative holographic navigation, and postoperative monitoring, could transform MR from an adjunct visualization tool into a comprehensive digital navigation framework.

Economic and usability analyses should also be incorporated to assess cost-effectiveness and learning curve dynamics. Furthermore, human–machine interaction deserves explicit attention as a clinical endpoint. MR fundamentally alters the surgeon's visual and cognitive environment; thus, quantitative assessment of cognitive load, ergonomics, attentional focus, and fatigue should accompany traditional surgical metrics. Only through multidisciplinary and multi-institutional collaboration will MR evolve from a promising innovation into a clinically validated, cost-effective, and scalable surgical technology.

## Conclusions

Mixed reality is redefining intraoperative visualization in orthopedic surgery, bridging the gap between digital preoperative planning and real-world execution. Its preferential application in complex hip and spine procedures reflects its clinical maturity in scenarios where three-dimensional orientation and millimetric accuracy are critical. The existing body of evidence primarily demonstrates technical feasibility and early clinical applicability of MR systems but does not yet provide sufficient high-quality evidence to support their clinical effectiveness or superiority over conventional surgical approaches.

Future validation requires well-designed prospective trials that quantify not only technical precision but also patient-centered outcomes, cost-effectiveness, and long-term functional results. Equally important will be the development of interoperable MR platforms capable of integrating seamlessly with existing navigation and robotic systems. Ultimately, the clinical translation of MR will depend on its ability to provide measurable improvements in safety, efficiency, and surgeon performance, positioning it as a cornerstone technology in the digital evolution of orthopedic surgery. Future studies must address the current methodological limitations, particularly the high risk of bias, through adequately powered randomized trials and standardized outcome reporting to enable reliable assessment of clinical benefit.

## Data Availability

The raw data supporting the conclusions of this article will be made available by the corresponding author, upon reasonable request.
